# Editorial: New advances in social influence: theoretical insights and methodological challenges

**DOI:** 10.3389/fpsyg.2023.1295390

**Published:** 2023-10-10

**Authors:** Antonis Gardikiotis, Stamos Papastamou, Gerasimos Prodromitis, William Crano

**Affiliations:** ^1^Department of Journalism and Mass Media Studies, Aristotle University of Thessaloniki, Thessaloniki, Greece; ^2^Department of Psychology, Panteion University, Athens, Greece; ^3^School of Social Science, Policy and Evaluation, Institute of Health Psychology and Prevention Science, Claremont Graduate University, Claremont, CA, United States

**Keywords:** social influence, minority influence, social cognition, climate change, dehumanization

Social influence is a central topic in social psychology, both as a field of research inquiry and as a fundamental process involved in a wide gamut of social psychological phenomena. Researchers and theorists in this field are interested in how people change their beliefs and attitudes by knowing that others (a majority or a minority social group) share a different opinion. The goal of this Research Topic is to present recent social influence research by focusing on theoretical advances, applications, and methodological issues.

In two articles of the Research Topic, social influence theorizing is applied to provide solutions to the pressing social question of how to improve individuals' thinking and behavior toward climate change.

In an experimental study, Seyranian et al. employ the *Context Comparison Model* (CCM) to develop persuasive communication concerning global climate change. They focus on attitudes and perceptions of the plausibility of climate change. CCM is a general model of social influence that considers the importance of target's attitudes (weak or strong), source status (ingroup or outgroup, majority or minority), and issue under consideration (subjective or objective) in the process of attitude change. They found that in terms of attitudes toward climate change (subjective task), communication coming from ingroup sources, and especially ingroup minorities, were more persuasive. In terms of plausibility of climate change (objective task), outgroup sources, and especially outgroup majority, had a persuasive advantage. These findings underline the complex social psychological processes affecting individuals' evaluations and perceptions of climate change. Those developing pro-environmental communications may be well-served by taking these complexities into account.

Avery and Butera also applied social influence theorizing to the climate crisis and asked whether minority messages that propose radical economic and societal changes could facilitate pro-environmentalism. They examined how exposure to counternormative pro-environmental minority messages (operationalized as messages advocating degrowth against the normative social dominant paradigm of continuous economic growth) could trigger different emotional reactions as a way of resisting the suggested change. Interestingly, they focused on the emotional reactions toward the message, a topic that has not been investigated systematically in the literature of social influence, and especially emotions with coping potential (control-oriented). A qualitative (employing semi-structured interviews) and a quantitative (employing online survey) study showed that participants reported higher control-oriented emotions (like anger and fear) than lower control-oriented emotions (like sadness). This suggests that the pro-environmental degrowth message is perceived as threatening and these emotional reactions help individuals to restore their support for the dominant paradigm of growth and thus resist change. Interestingly, this pattern of results was more pronounced among men, who are presumably socialized to maintain the status quo and preserve their status in society.

In their study, Quiamzade and Lalot take a novel approach by studying the topic of dehumanization (usually found in the intergroup relations literature) as an influence process. They test the intriguing idea that animalistic dehumanization of a minority group (Roma beggars) could function as a social influence strategy that would immunize individuals toward subsequent favorable messages toward the minority group. Participants initially received one of three possible initial messages depicting minority groups in a manner that was negative animalized, negative humanized, or positive humanized. Then, all participants received a second message advocating for minority rights. Although both negative animalized and negative humanized conditions led to negative attitudes and discriminatory behavioral intentions toward the minority, it was only in the latter condition that these effects persisted even after exposure to favorable messages advocating for the rights of the minority group. Animalistic dehumanization of the minority group thus seems to function as an influence strategy immunizing individuals against the influence of the subsequent positive message. These findings could be useful in understanding the lasting effects of pejorative and discriminatory discourse against minority groups in our societies, even in the face of positive pro-minority advocacy.

Linne et al. position their work within the paradigm of social cognition and examine the dynamic nature of information processing in persuasion. They focus particularly on the specific sequence of processing information during an attitude change attempt leading to assimilation or contrast outcomes. They propose the model of sequential information processing whereby inferences drawn from initial exposure to information may bias the processing of subsequent information. Results suggest that the sequence of the same arguments determines the emergence of assimilation or contrast outcomes depending on the processing of the valence of information. These initial results provide useful insights for persuasion by two-sided messages, with strong contrasting arguments being more persuasive when introduced after unambiguous contrary arguments.

Finally, Prislin lays out a revitalizing agenda for future research in minority influence, where attention should be paid to social change itself and not only on the cognitive processes and attitudinal changes induced by minorities, which have occupied the lion's share of social influence literature to date. Prislin proposes three important conceptual and empirical points. The first is that influence unravels over time and important questions remain about constructions of influence over time, how attitudes change evolves for message recipients, and how it spreads among groups. The second concerns the dynamic nature of influence. Factions within and between groups compete to advance their positions. Gaining or losing social support due to successful or failed influence has reciprocal effects on both sources of influence. The third concerns the motives behind minority influence. Social change is pursued because minorities: (a) seek social validation, having a sense of correctness that motivates them to exert influence, (b) seek social acceptance, striving for social belonging and acceptance of their distinct identity, and (c) seek social control, endeavoring to access tangible benefits (e.g., resources, power). These points provide a useful basis for advancing social influence research.

The articles of this Research Topic contribute to the current social influence research by dealing with real life and diverse social phenomena (such as climate change, dehumanization), the underlying cognitive processes which foster development of a research agenda for the future, which if adopted, could not help but benefit society.

## Author contributions

AG: Writing—original draft. SP: Writing—review and editing. GP: Writing—review and editing. WC: Writing—review and editing.

